# Initial experiences of switching to faricimab for neovascular age-related macular degeneration and polypoidal choroidal vasculopathy in an Asian population

**DOI:** 10.3389/fopht.2023.1346322

**Published:** 2024-01-11

**Authors:** Farah N. I. Ibrahim, Kelvin Y. C. Teo, Tien-En Tan, Hiok Hong Chan, Priya R. Chandrasekaran, Shu-Yen Lee, Anna C. S. Tan, Ranjana Mathur, Choi Mun Chan, Shaun S. Sim, Gavin Siew Wei Tan, Ian Y. S. Yeo, Chui Ming Gemmy Cheung

**Affiliations:** ^1^ Medical Retina Department, Singapore National Eye Centre, Singapore, Singapore; ^2^ Duke-NUS Medical School, National University of Singapore, Singapore, Singapore; ^3^ Save Sight Institute, University of Sydney, Sydney, NSW, Australia

**Keywords:** ANG-2, anti-vegf, faricimab, neovascular age-related macular degeneration, polypoidal choroidal vasculopathy, switch therapy

## Abstract

**Purpose:**

To describe the early experiences of patients with neovascular age-related macular degeneration (nAMD) and polypoidal choroidal vasculopathy (PCV) whose treatment was switched to faricimab from other anti-vascular endothelial growth factor (VEGF) agents.

**Methods:**

This is a prospective cohort of eyes with nAMD and PCV that were previously treated with anti-VEGF agents other than faricimab. We evaluated visual acuity (VA), central subfield thickness (CST), macular volume (MV), pigment epithelial detachment (PED) height, and choroidal thickness (CT) after one administration of faricimab. Where present, fluid was further evaluated according to intraretinal fluid (IRF), subretinal fluid (SRF), or within PED.

**Results:**

Seventy-one eyes from 71 patients were included (45.07% PCV and 54.93% typical nAMD). The mean [standard deviation (± SD)] VA, CST, and MV improved from 0.50 logMAR (± 0.27 logMAR) to 0.46 logMAR (± 0.27 logMAR) (*p* = 0.20), 383.35 µm (± 111.24 µm) to 322.46 µm (± 103.89 µm (*p* < 0.01), and 9.40 mm^3^ (± 1.52 mm^3^) to 8.75 mm^3^ (± 1.17 mm^3^) (*p* < 0.01) from switch to post switch visit, respectively. The CT reduced from 167 µm (± 151 µm) to 149 µm (± 113 µm) (*p* < 0.01). There was also a significant reduction in the maximum PED height between visits [302.66 µm (± 217.97 µm)] and the post switch visit [236.66 µm (± 189.05 µm); *p* < 0.01]. This difference was greater in PEDs that were predominantly serous in nature. In the eyes with typical nAMD (*n* = 39), improvements were significant for CST, MV, CT, and PED. In the eyes with PCV (*n* = 32), only reductions in CT were statistically significant, while VA, CST, MV, and PED only showed numerically smaller improvements. One patient developed mild vitritis without vasculitis, which resolved with topical steroids with no sequelae.

**Conclusions:**

In our case series of Asian nAMD patients, switching to faricimab was associated with a stable VA and meaningful anatomical improvements, particularly with typical nAMD subtypes.

## 1 Introduction

Current vascular endothelial growth factor (VEGF) inhibitors are very effective in limiting the detrimental effects of neovascular age-related macular degeneration (nAMD) and polypoidal choroidal vasculopathy (PCV) (1–3). However, frequent retreatment is often necessary. Furthermore, exudative activity cannot be fully controlled in a subgroup of patients despite having frequent injections (4, 5). There is, hence, an unmet need in today’s treatment paradigm in addressing this group of patients.

Faricimab is the newest intravitreal therapy approved for the treatment of nAMD. Its mechanism of action is bispecific, providing both VEGF-A and angiopoietin 2 (ANG-2) inhibition. The bispecific action has been proposed to control neovascularization, enhance vessel stability, and provide increased durability (6). The recently concluded registration trials have demonstrated a durability of effect, with almost 80% of participants achieving retreatment intervals of 12 weeks or longer (7). Although the TENAYA and LUCERNE trials have established the efficacy of faricimab in treatment-naive eyes under clinical trial conditions, few trials have reported the performance of faricimab in real-world clinical practice. As current anti-VEGF agents are able to control the disease process and maintain visual acuity (VA) well, the first faricimab experience for many may be in the context of patients that do not respond well to the current treatments.

Another limitation of the TENAYA and LUCERNE trials is the limited representation of non-white populations. Participants of Asian descent constituted about 10% of the TENAYA and LUCERNE study population. While the analysis of this subgroup reported results consistent with the overall study population, further clinical experience is urgently needed to understand the response in Asian patients. This is due to the differences in the AMD phenotype in Asian patients, specifically, the high prevalence of polypoidal choroidal vasculopathy (PCV) in this population. The response of PCV to anti-VEGF agents has been shown in some studies to be more heterogeneous than typical nAMD (8), and the potential benefit of ANG-2 inhibition in PCV is uncertain. Due to the low prevalence of PCV in the TENAYA and LUCERNE trials, the efficacy and safety of faricimab in PCV will be evaluated in the recently initiated SALWEEN study (NCT03823287).

In this report, we describe the first experience of the use of faricimab in Asian patients with nAMD and PCV who were previously treated with other anti-VEGF agents.

## 2 Materials and methods

This study conformed to the tenets of the Declaration of Helsinki and was approved by the international review board and by the ethics committee. Written informed consent was provided by all participants before participation in the study for the Singapore National Eye Centre (R1921/49/2022). Faricimab (Vabysmo™; Roche) became commercially available in Singapore from 16 June 2022.

### 2.1 Study population

In this analysis, we prospectively enrolled patients with typical nAMD and PCV who were switched to faricimab after a period of treatment with other anti-VEGF agents in the Singapore National Eye Centre. The reason for switching and the follow-up interval post switch was not standardized. However, patients were offered faricimab therapy if there was persistently active disease despite there being regular retreatment intervals. Multimodal imaging, including optical coherence tomography (OCT), fluorescein fundus angiography, and indocyanine green angiography, was performed on initial diagnosis in all cases.

### 2.2 Outcome measures

The main outcomes of interest were change in VA and several OCT-based anatomical parameters between the time of switching and the post-switch assessment. The visit at which faricimab was first administered was defined as the “switch visit” and the visit immediately after was defined as the “post-switch visit”. After the first faricimab injection, most patients were reviewed at an interval of ± 2 weeks from the treatment interval prior to switching.

The functional outcome measure was the VA as measured by logMAR. VA stability was defined as maintaining VA of ± 1 line logMAR from the switch visit. The anatomical outcomes included qualitative evaluation of the fluid compartments according to intraretinal fluid (IRF), subretinal fluid (SRF), or pigment epithelial detachment (PED). These features were graded on OCT by a retinal specialist and a retinal fellow, and were classified as complete resolution, partial resolution, unchanged, or worse between the two visits. The presence of PED was identified as a separation between the retinal pigment epithelium (RPE) and Bruch’s membrane (BM) of at least 100 µm (9). The PED was also subdivided in those eyes that were predominantly serous in nature (≥ 50% homogenously hyporeflective) or not (< 50% homogenously hyporeflective).

The quantitative measures on OCT also included central subfield thickness (CST), macular volume (MV), maximum PED height, and subfoveal choroidal thickness (CT). The CST and MV measurements in the central 1 mm and 6 mm Early Treatment of Diabetic Retinopathy Study (ETDRS) grid centered on the fovea, respectively, were evaluated. The maximum height of the PED was measured at the OCT cut, with the highest PED as the distance from the superior border of the RPE to the base of the Bruch’s membrane (BM). CT was obtained by measuring the distance from the base of the BM to the chorio–scleral interface at the fovea. All the measurements were performed using built-in software or manual calipers on the Heidelberg Explorer (HEYEX), Spectralis OCT platform. In addition, manual correction of the relevant segmentation was performed, if necessary.

### 2.3 Statistical methods

The categorical variables were presented as the number and percentage of patients in each category. The continuous variables were summarized using descriptive statistics [mean, standard deviation (SD), median, and interquartile range (IQR)], as appropriate. The descriptive statistics were provided for patient demographics, baseline characteristics, treatment course, and clinical characteristics at the switch visit. The Wilcoxon rank-sum test was applied to all paired comparisons (between the post switch and the switch visits) taking a *p*-value of < 0.05 as a level of statistical significance. All the statistics were performed with R, version 4.2.2 (The R Foundation for Statistical Computing, Vienna, Austria).

## 3 Results

### 3.1 Overall patient demographics and clinical characteristics

Seventy-one Asian patients with nAMD (mean ± SD age of 70.6 years ± 8.0 years; 56.34% male) were enrolled in the study. A total of 39 eyes had typical nAMD [38 (53.52%) type 1 and one (1.41%) type 2 MNV] and 32 had PCV (45.07%). The mean (± SD) age of patients with PCV and typical nAMD was 67.7 years (± 7.8 years) and 74.8 years (± 6.7 years), respectively. The treatment history was comprised of a mean (± SD) of 23.02 (± 16.53) anti-VEGF injections per eye over a mean (± SD) period of 40.19 months (± 30.26 months) in the overall cohort. Among the patients with typical nAMD, the treatment history comprised a mean (± SD) of 19.61 (± 12.03) anti-VEGF injections over a mean (± SD) period of 35.38 months (± 27.2 months). The eyes with PCV had received a mean of (± SD) 26.08 (± 19.43) anti-VEGF injections over a mean (± SD) period of 45 months (± 32.99 months). Immediately prior to switching to faricimab, 61.1% received aflibercept, 37% received ranibizumab, and 1.9% received bevacizumab. The mean (± SD) treatment interval pre switch was 7.83 weeks (± 5.41 weeks) in the overall cohort. The mean (± SD) treatment intervals pre switch for the eyes with typical nAMD and PCV were 6.5 weeks (± 2.24 weeks) and 6.79 weeks (± 3.8 weeks), respectively. The distribution of treatment intervals was 73.3%, 15%, and 11.7% in the ≤ 8-week, 9- to 12-week, and > 12-week intervals, respectively.

At the time of switching, the overall mean (± SD) VA was 0.5 logMAR (± 0.27 logMAR) with a CST of 383.35 µm (± 111.24 µm). The MV was 9.40 mm^3^ (± 1.52 mm^3^), the maximum PED height was 302.66 µm (± 217.97 µm), and the CT was 187 µm (± 105 µm). All the patients had fluid present on OCT at the time of switching: SRF only (67.6%), IRF only (11.3%), and both SRF and IRF present (21.1%). **Table 1** summarizes the characteristics of the patients who received faricimab therapy.

**Table 1:** Demographics and the clinical characteristics of patients with nAMD and PCV who received faricimab therapy.

**No. of eyes, n**	71
**Age, mean (SD)**	70.6 (± 8.0)
**Gender, Female (%)**	Male 40 (56.34%), Female 31 (43.66%)
**Diagnosis**	
Type 1 MNV, n (%)	38 (53.52%)
Type 2 MNV, n (%)	1 (1.41%)
PCV, n (%)	32 (45.07%)
Duration of anti-VEGF at switch, months	
Overall cohort (n=71)	
Mean (SD)	40.19 (± 30.26)
Median (IQR)	35.5 (28.8)
Range	4-137
Eyes with typical nAMD (n=39)	
Mean (SD)	35.38 (± 27.2)
Median (IQR)	26 (35)
Range	4-114
Eyes with PCV (n=32)	
Mean (SD)	45 (± 32.99)
Median (IQR)	35 (23)
Range	4-137
Number of anti-VEGF at switch, n	
Overall cohort	
Mean, (SD)	23.02 (± 16.53)
Median, (IQR)	20.5 (16.5)
Range	4-73
Eyes with typical nAMD	
Mean, (SD)	19.61 (± 12.03)
Median, (IQR)	17 (19)
Range	4-46
Eyes with PCV	
Mean, (SD)	26.08 (± 19.43)
Median, (IQR)	21 (16.5)
Range	4-73
Retreatment interval immediately before switch (weeks)	
Overall cohort	
Mean, (SD)	7.83 (± 5.41)
Median (IQR)	6 (3)
Range	4- 12
Eyes with typical nAMD	
Mean (SD)	6.5 (± 2.24)
Median (IQR)	7 (4)

nAMD, neovascular age-related macular degeneration; MNV, macular neovascularisation; PCV, polypoidal choroidal vascularisation; VEGF, vascular endothelial growth factor; SD, standard deviation; IQR, interquartile range; OCT, optical coherence tomography; SRF, subretinal fluid; IRF, intraretinal fluid; CT, choroidal thickness.

### 3.2 Visual and anatomical outcomes in the overall cohort

**Figure 1:**
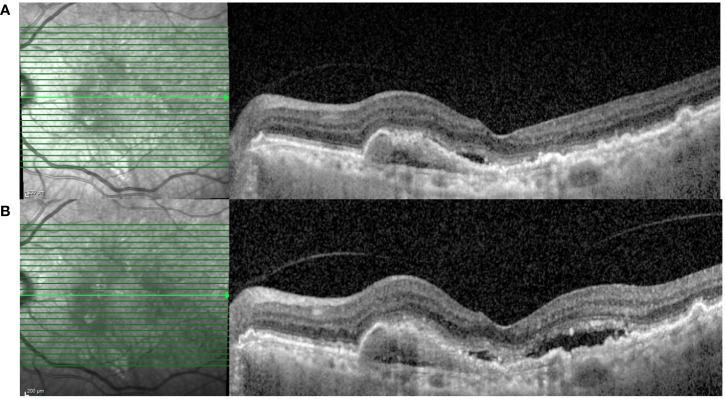
A 83-year-old male with type 1 MNV was switched to faricimab after persistent SRF and a large PED despite having five-weekly injections of ranibizumab. **(A)** SD-OCT at the pre-switch visit (VA: 0.3 logMAR; CST: 276 µm; and MV: 8.11 mm^3^). **(B)** SD-OCT after the first faricimab injection demonstrating increased subretinal fluid (VA: 0.54 logMAR; CST 338 µm; and MV: 8.6 mm^3^). MNV, macular neovascularisation; SRF, subretinal fluid; PED, pigment epithelial detachment; SD-OCT, spectral domain optical coherence tomography; VA, visual acuity; CST, central subfield thickness; MV, macular volume.

At the first post-switch visit, which took place at a mean (± SD) interval of 6.72 weeks (± 2.86 weeks) post injection, 97.19% patients had a stable (± 1 line) or improved VA, compared with the switch visit. Two eyes (2.81%) had a worsening VA of between 2 logMAR lines and 3 logMAR lines after the first faricimab injection, with an increased activity on OCT (**Figure 1**). Among the two eyes, one eye with PCV had previously received 49 anti-VEGF injections over 84 months and one eye with T1 MNV had received 12 anti-VEGF injections over 12 months. None of these eyes developed any new retinal hemorrhages.

Overall, the mean (± SD) VA was better at the post switch [0.46 logMAR (± 0.27 logMAR)] than at the switch visit [0.50 logMAR (± 0.27 logMAR); *p* = 0.20]. Likewise, the CST [mean (± SD) 332.46 µm (± 103.89 µm) vs. 383.35 µm (± 111.24 µm); *p* < 0.01] and MV [8.75 mm^3^ (± 1.7 mm^3^) vs. 9.40 mm^3^ (± 1.52 mm^3^); *p* < 0.01] were significantly lower at the post-switch visit. Similarly, the maximum PED height [236.66 µm (± 189.05 µm) vs. 302.66 µm [± 217.97 µm; *p* = 0.02] and CT [149 µm (± 113 µm) vs. 167 µm (± 151 µm); *p* < 0.01) at post switch was significantly less that at the switch visit. **Table 2** outlines the difference in baseline characteristics and outcomes in the overall cohort.

**Table 2:** Baseline characteristics and the outcome measures in the overall cohort and stratified by lesion subtype.

	All eyes (n=71)			typical nAMD (n=39)			PCV (n=32)		
	At switch	Post switch	P value*	At switch	Post switch	P value*	At switch	Post switch	P value*
Mean age, years (SD)	70.6 (8.0)			74.8 (6.7)			67.7 (7.8)		
Mean VA, LogMAR (SD)	0.50 (0.27)	0.46 (0.27)	0.20	0.45 (0.24)	0.43 (0.26)	0.35	0.56 (0.30)	0.51 (0.29)	0.21
Mean CST, µm (SD)	383.35 (111.24)	332.46 (103.89)	<0.01	396.35 (112.63)	325 (110.99)	<0.01	367.50 (109.17)	341.56 (95.48)	0.15
Mean macular cube volume, mm3 (SD)	9.40 (1.52)	8.75 (1.17)	<0.01	9.50 (1.45)	8.65 (1.29)	<0.01	9.28 (1.61)	8.88 (1.01)	0.12
Mean maximum PED height, µm (SD)	302.66 (217.97)	236.66 (189.05)	0.027	291.07 (156.84)	226.38 (137.83)	0.025	316.78 (279.03)	249.18 (239.92)	0.15
Mean CT, µm (SD)	167 (151)	149 (113)	<0.01	160 (122)	146 (128)	0.03	187 (140)	143 (142)	0.01

PCV, polypoidal choroidal vascularisation; nAMD, neovascular age related macular degeneration; VA, visual acuity; CST, central subfield thickness; PED, pigment epithelial detachment; CT, choroidal thickness; SD, standard deviation.

* P value is calculated by wilcoxan rank sum comparing measurements between switch and post switch visits.

**Figure 2:**
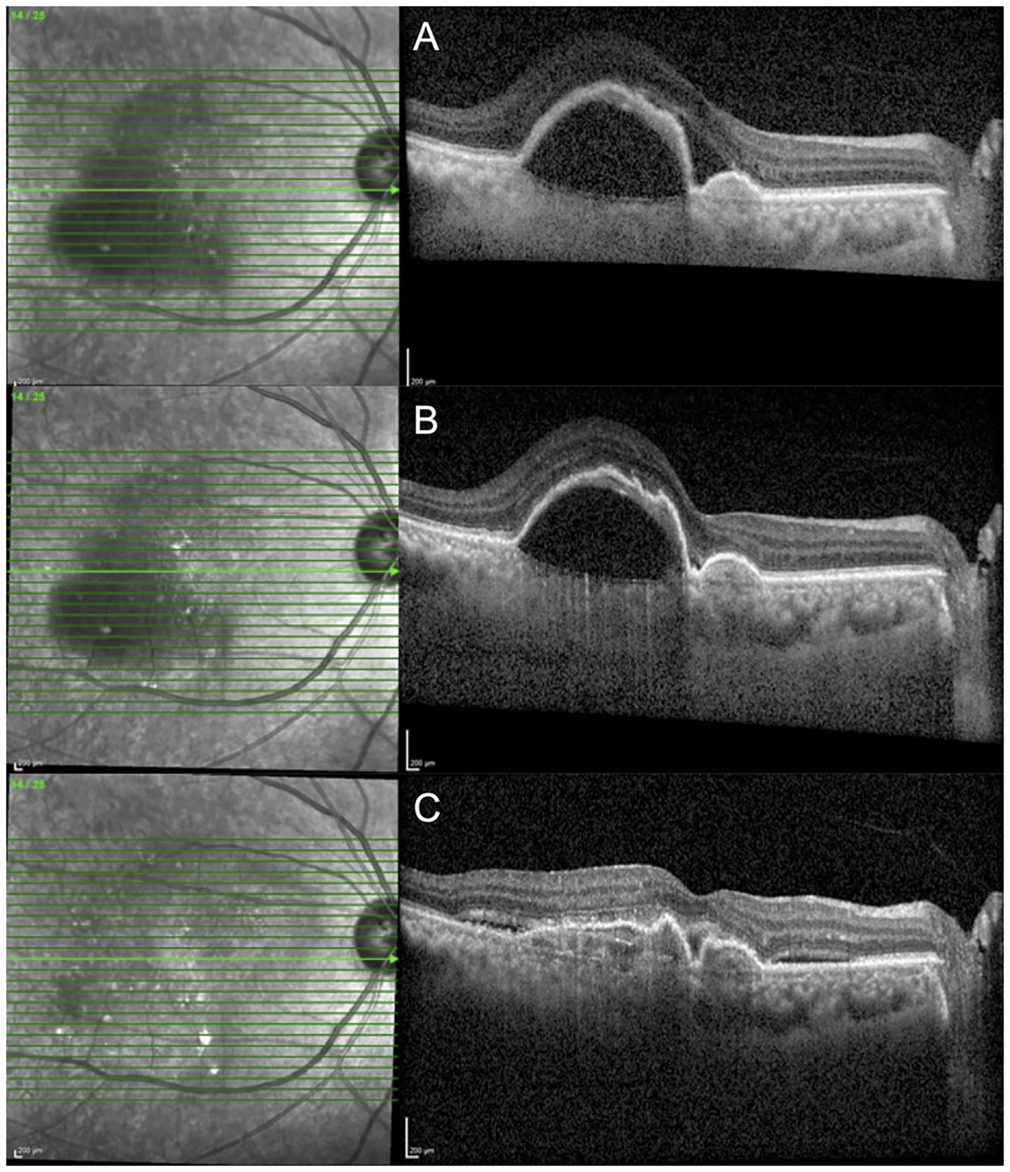
A 65-year-old female with nAMD who was switched to faricimab after demonstrating persistent SRF and a large PED despite having six-weekly injections of aflibercept. **(A)** SD-OCT at the pre-switch visit despite having six weekly injections of aflibercept (VA: 0.3 logMAR; CST: 554 µm; and MV: 13.18 mm^3^). **(B)** SD-OCT demonstrating resolution of SRF after the first faricimab injection (VA: 0.3 logMAR; CST: 428 µm; and MV: 11.01 mm^3^). **(C)** SD-OCT demonstrating a significant improvement in PED after the second and third faricimab injection; [VA: 0.4 logMAR; CST: 351 µm; and MV: 8.81 mm^3^). nAMD, neovascular age related macular degeneration; SRF, subretinal fluid; PED, pigment epithelial detachment; SD-OCT, spectral domain optical coherence tomography; VA, visual acuity; CST, central subfield thickness; MV, macular volume.

**Figure 3:**
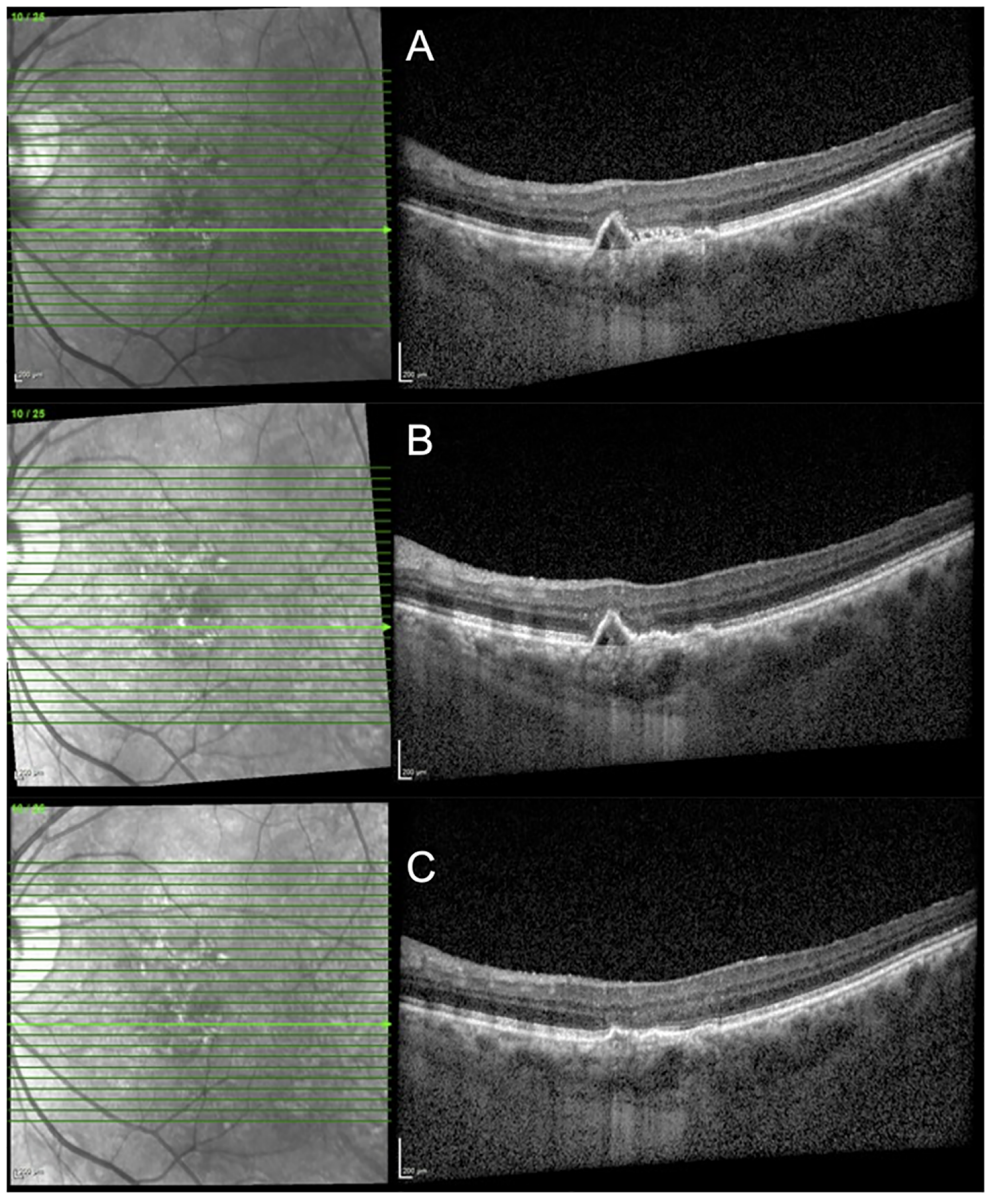
A 71-year-old patient with PCV had persistent SRF despite having multiple regular 10-weekly injections of aflibercept. **(A)** SD-OCT at the pre-switch visit (VA: 0.3 logMAR; CST: 272 µm; and MV: 8.34 mm^3^). **(B)** SD-OCT demonstrating resolution of SRF at 8 weeks after the first faricimab injection (VA: 0.2 logMAR; CST: 257 µm; and MV: 8.21mm^3^). **(C)** SD-OCT demonstrating flattening of the PED after two faricimab injections at 10-weekly intervals (VA: 0.1 logMAR; CST: 254 µm; and MV: 8.16 mm^3^). PCV, polypoidal choroidal vasculopathy; SRF, subretinal fluid; PED, pigment epithelial detachment; SD-OCT, spectral domain optical coherence tomography; VA, visual acuity; CST, central subfield thickness; MV, macular volume.

**Figure 4:**
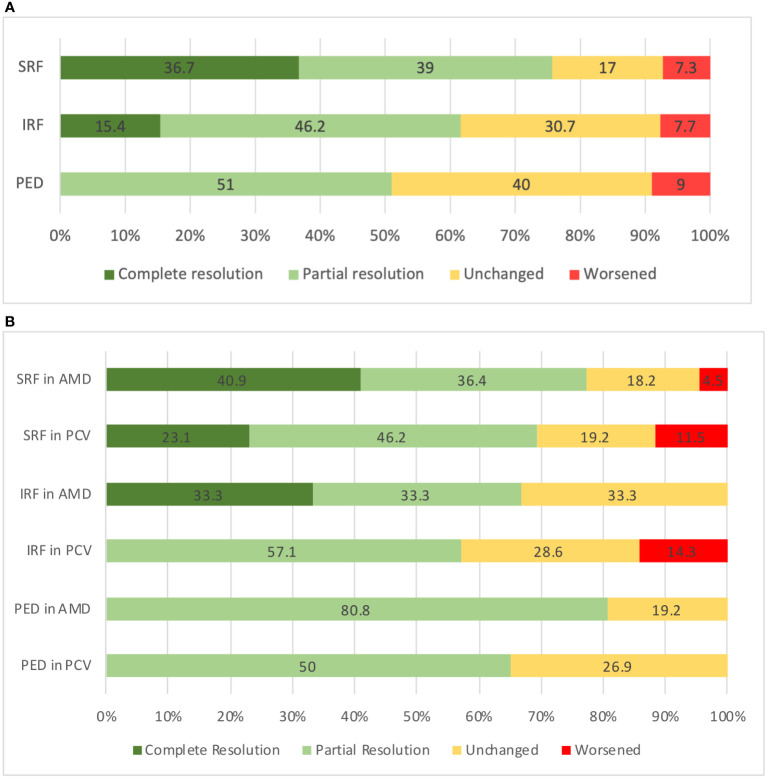
**(A)** Status at the follow-up visit according to the fluid compartment in all eyes (*n* = 71). **(B)** Status at the follow-up visit according to the fluid compartment as stratified by lesion type. SRF, subretinal fluid; IRF, intraretinal fluid, PED, pigment epithelial detachment; AMD, age related macular degeneration; PCV, polypoidal choroidal vasculopathy.

A total of 51 eyes (71.8%) demonstrated at least partial resolution of both SRF and IRF; out of which 23 eyes (32.3%) achieved complete resolution of both SRF and IRF after a single injection. The cases of structural response to faricimab treatment are demonstrated in **Figures 2**, **3**. The detailed distribution of patients with fluid within different retinal compartments at the follow-up visit is shown in **Figure 4A**. We further divided the PED according to the reflectivity of their content. A total of 29 (40.84%) eyes were graded as predominantly serous and a reduction in the maximum PED height was significant in this group [mean (± SD) −130.31 µm (± 132.58 µm); *p* < 0.01). This is in contrast to the 42 (59.16%) eyes that had PED graded as not predominantly serous, where the mean (± SD) maximum PED height reduction was −21.59 µm (± 38.18 µm; *p* = 0.23).

When comparing the outcomes between those eyes that received ranibizumab (*n* = 25) or aflibercept (*n* = 40) immediately prior to the switch, the mean (± SD) change in the VA, CST, MV, and maximum PED height were −0.03 logMAR (± 0.14 logMAR) versus −0.04 logMAR (± 0.11 logMAR) (*p* = 0.42), −63.6 µm (± 129.54 µm) versus −44.47 µm (± 109.61 µm) (*p* = 0.27), 0.87 mm^3^ (± 1.67 mm^3^) versus 0.58 mm^3^ (± 1.36 mm^3^) (*p* = 0.20) and −90.48 µm (± 120.03 µm) versus −57.9 µm (± 97.47 µm), (*p* = 0.12), respectively. **Table 3** describes the outcome measures of the patients who were switched from ranibizumab or aflibercept to faricimab.

**Table 3:** Outcome measures in the eyes that received ranibizumab or aflibercept injections immediately prior to switching to faricimab.

	Ranibizumab (n=25)	Aflibercept (n= 40)	P value*
Mean change in VA, LogMAR (±SD)	-0.03 (±0.14)	-0.04 (±0.11)	0.42
Mean change in CST, µm (±SD)	-63.6 (±129.54)	-44.47 (±109.61)	0.27
Mean change in macular cube volume, mm3 (±SD)	0.87 (±1.67)	0.58 (±1.36)	0.20
Mean change in maximum PED height, µm (±SD)	-90.48 (±120.03)	-57.9 (±97.47)	0.12

VA, visual acuity; CST, central subfield thickness; PED, pigment epithelial detachment; SD, standard deviation.

* P value is calculated by wilcoxan rank sum comparing measurements between switch and post switch visits.

Among the 71 eyes, 45 (63.38%) received three consecutive faricimab injections at a matched interval prior to the switch. In this group (45.5% PCV; 54.5% nAMD), significant reductions in CST, MV, and PED [CST mean (SD): −69.44 µm (± 24 µm), *p* < 0.01; MV mean (SD): −0.81 mm^3^ (± 0.38 mm^3^), *p* < 0.01; and PED mean (SD): −149.67 µm (± 138 µm; *p* < 0.01] were observed. The mean (SD) VA was better after three faricimab injections [0.4529 logMAR (± 0.29 logMAR)], compared with the switch visit [0.48 logMAR (± 0.27 logMAR); *p* = 0.30]. **Table 4** outlines the difference in the baseline characteristics and the outcomes in the eyes that received the three consecutive faricimab injections.

**Table 4:** Baseline characteristics and the outcome measures in the eyes that received three faricimab injections.

	Eyes which received three consecutive faricimab injections (n=45)			
	At switch	Post faricimab #1	Post faricimab #3	P value*
Mean VA, LogMAR (SD)	0.48 (0.27)	0.45 (0.25)	0.45 (0.29)	0.30
Mean CST, µm (SD)	387.44 (126.23)	319.37 (97.15)	318 (83.14)	<0.01
Mean macular cube volume, mm3 (SD)	9.48 (1.69)	8.68 (1.23)	8.67 (1.14)	<0.01
Mean maximum PED height, µm (SD)	334.22 (253)	250 (220.79)	184.55 (116.82)	<0.01

VA, visual acuity; CST, central subfield thickness; PED, pigment epithelial detachment; SD, standard deviation.

* P value is calculated by wilcoxan rank sum comparing measurements between switch and post Faricimab #3 visits.

### 3.3 Matched-interval sensitivity analysis

We also analyzed the outcomes in 37 patients who were reviewed at a matched interval prior to the switch. This group of patients was previously treated at a mean (± SD) interval of 5.83 weeks (± 1.53 weeks) prior to the switch. In this cohort, the mean (± SD) VA, CST, and MV at the time of switch were 0.48 logMAR (± 0.25 logMAR), 373.54 µm (± 94.9 µm), and 9.21 mm^3^ (± 1.29 mm^3^), respectively. At the first post-switch visit, which took place at mean (± SD) interval of 5.54 weeks (± 1.4 weeks), the VA improved by a mean (± SD) of 0.05 logMAR (± 0.15 logMAR; *p* = 0.04). A mean (± SD) reduction in CST and MV of 41.14 µm (± 81.2 µm; *p* ≤ 0.01) and 0.53 mm^3^ (± 1.16 mm^3^); *p* ≤ 0.01, respectively, was also observed.

### 3.4 Visual and anatomical outcomes of the eyes stratified by lesion type

In the eyes with typical nAMD subtypes (i.e., types 1 and 2 MNV), the VA was better at the post switch than at the switch visit, [mean (± SD) 0.43 logMAR (± 0.26 logMAR) vs. 0.45 logMAR (± 0.24 logMAR); *p* = 0.35). Likewise, the CST, [325 µm (± 110.99 µm) vs. 396.35 µm (± 112.63 µm); *p* < 0.01] and MV [8.65 mm^3^ (± 1.29 mm^3^) vs. 9.50 mm^3^ [± 1.45 mm^3^); *p* < 0.01) were significantly reduced at the post switch visit compared with the switch visit. The maximum PED height [226.38 µm (± 137.83 µm) vs. 291.07 µm [± 156.84 µm); *p* = 0.02] and CT (146 µm (± 128 µm) vs. 160 µm (± 122 µm); *p* = 0.03) post switch were also lower than at the switch visit. Among the 22 eyes with SRF at the switch visit, nine (40.9%) achieved complete resolution, eight (36.4%) achieved partial resolution, four (18.2%) remained unchanged, and one (4.5%) worsened. Of the six patients with IRF at the time of the switch, two (33.3%) had complete resolution, two (33.3%) achieved partial resolution and three (33.3%) remained unchanged. Among the 26 eyes with PED at the switch visit, 21 (80.8%) had partial resolution, whereas five (19.3%) remained unchanged.

In the eyes with PCV, only [mean (± SD)] the CT was significantly reduced in the post-switch visit [143 µm (± 142 µm) vs. 187 µm (± 140 µm); *p* = 0.01] compared with the switch visit. Of all the eyes with SRF, six (23.1%) eyes achieved complete resolution of SRF after the faricimab injection, 12 (46.2%) achieved partial resolution, five (19.2%) remained unchanged and three (11.5%) worsened. Among the seven eyes with IRF, four (57.1%) had partial resolution, two (28.6%) remained unchanged, whereas one (14.3%) worsened. A total of 13 (50%) eyes had partial resolution of PED, seven (26.9%) remained unchanged, and three (23.1%) worsened. **Figure 4B** demonstrates the status at the follow-up visit according to fluid compartment in PCV and typical nAMD patients. **Table 2** outlines the difference in baseline characteristics and the outcomes between typical nAMD and PCV subgroups.

### 3.5 Adverse events

One patient developed intraocular inflammation 8 weeks post treatment with faricimab, which was suspected to be drug related. The patient reported mild eye redness 8 weeks post injection, and anterior chamber cells 1+ and vitritis 1+ were noted at that the follow-up visit. No evidence of retinitis or vasculitis was present. The inflammation completely resolved after treatment with topical steroids (1% prednisolone acetate four times a day for 14 days, followed by tapering dose over 21 days). Intravitreal aflibercept was subsequently resumed, and no deterioration of VA was observed; the VA was maintained at 0.4 logMAR at the switch and post-switch visit. No other ocular adverse events including endophthalmitis, retinal vasculitis, retinal detachment, retinal artery occlusions, or sustained elevated IOP requiring topical ocular hypertensive mediations were observed. Systemic complications, including thromboembolic events such as myocardial infarction or cardiovascular accident, were not observed during the study period.

## 4 Discussion

This case series summarizes an early real-world experience of switching to faricimab from other anti-VEGF agents in eyes with nAMD and PCV in an Asian population (10). Overall, we report stable or improved vision in the majority of switched eyes and an anatomical improvement in a proportion of switched eyes. Despite the significant change in the structural outcomes, the improvement in VA was small, if present. The disconnect between morphology and function has been reported before in other switch studies (11–13). The longstanding ocular history of nAMD in our cohort may contribute to the limited potential of visual recovery due to underlying chronic structural damage. This is reflected in the large number of patients in this study who had previously received aggressive treatment with other anti-VEGF agents (with a mean number of 23 injections) prior to switching to faricimab.

Faricimab is the first bispecific monoclonal antibody designed for intraocular use via intravitreal injection, which independently binds and neutralizes both ANG-2 and VEGF-A, enabling dual inhibition of two distinct pathways involved in the nAMD pathology (14). Data from completed phase 3 studies demonstrated sustained efficacy and extended durability of faricimab (7). In the current series, we included eyes with persistent fluid whose treatment was switched to faricimab from other anti-VEGF agents. Overall, we observed encouraging outcomes, including an anatomical improvement in 83.7%, and 27% gained ≥ 2 lines of logMAR VA. Although the first post-switch visit (6.7 weeks) was shorter (not statistically significant) than the pre-switch treatment interval (7.8 weeks), the results in a small matched-interval cohort were found to be consistent with the overall cohort. Similarly, early real-world data for faricimab in the TRUCKEE trial have also shown promising outcomes for VA, safety, and central subfoveal thickness in both treatment-naive and previously treated patients (15). Our analysis of the fluid compartment adds further to our understanding of the effect of faricimab; SRF and IRF responded well with over 70% exhibiting at least partial resolution, and 36.7% (SRF) and 15.4% (IRF) exhibiting a complete resolution. It is well recognized that sub-RPE fluid is less responsive to anti-VEGF therapies than SRF or IRF. In patients with PED, we observed that 51% showed a partial response, whereas 40% and 9% remained unchanged or worsened, respectively, in our cohort. Interestingly, however, when the content of the PED was taken into consideration, we observed a marked reduction in the PED height in eyes with predominantly serous PED. This suggests that faricimab may have a beneficial effect on “sub-RPE fluid”. The reduction in the PED height could be a result of a reduction in leakage and subsequent resorption of fluid from anti-VEGF therapy, as well as a contraction of fibrocytes and other inflammatory cells, which has previously been described in eyes which have received multiple anti-VEGF injections (16). We hypothesize that the synergistic effects of ANG-2 and VEGF inhibition by faricimab further decreases vascular leakage and inflammation, reduces vascular RPE permeability, thus enhancing its effects in treating “sub-RPE fluid” (17).

Due to the small number of participants with PCV in the TENAYA and LUCERNE trials, there remains a very limited understanding of the potential action of faricimab on PCV eyes. PCV has been reported to be more prevalent in Asians than in Caucasians, and the distinct differences in pathophysiological and clinical factors may explain the more heterogeneous response to anti-VEGF treatment compared with typical nAMD (18). The efficacy and safety of faricimab for the treatment of PCV will be evaluated in the SALWEEN study, which is currently enrolling (NCT03823287). In this case series, the patients with PCV were younger (with a mean age of 67 years vs. 74 years) and had a lower mean CST at the time of switching than the typical nAMD group. We observed no change in the VA or MV, and small and non-significant reductions in the CST and maximum PED height in the switch/post-switch comparisons in PCV eyes, whereas switch/post-switch improvements in eyes with typical nAMD were numerically greater and achieved statistical significance. Nevertheless, in the PCV group, 24 out of 32 eyes with SRF and 19 out of 32 eyes with PED showed at least partial resolution after switching to faricimab. Overall, however, there was a significant reduction in CT in all nAMD subtypes suggesting that faricimab may have role in pachychoroid conditions. Inoda et al. found that the levels of ANG-2 were elevated in eyes with pachychoroid features, indicating the role of angiopoietins in choroidal remodeling (17). Our results suggest that dual inhibition of ANG-2 and VEGF-A enhances choroidal vascular remodeling in Asian patients with nAMD, particularly for those with a pachychoroid phenotype.

The strengths and limitations of this study should be considered. This is one of the first real-world studies to report outcomes of switching in an Asian population and it also included a subgroup of eyes with PCV. A Japanese multicenter study by Mukai et al. demonstrated a visual and anatomical improvement in nAMD and PCV eyes following faricimab therapy (19). Although the Japanese cohort included findings in treatment-naive eyes after three consecutive monthly injections of faricimab, our study focused on the outcomes in eyes that were switched to faricimab after previously being treated with other anti-VEGF agents. The limitations of the study also need to be considered. In this study we did not assess the best corrected VA. Although a pre- and post-switch comparison was performed in each of the typical nAMD and PCV subgroups, no direct between-group comparisons were performed due to the small number of patients in this study. In PCV eyes, no repeat ICGA was performed to assess polypoidal lesion regression, as we focused our evaluation on changes in fluid compartments. We also focused on the observations after a single faricimab injection. It is possible that more impressive improvements could be achieved with monthly loading or after a course of several faricimab injections. The patients also had different prior anti-VEGF agents and regimens and various reasons for switching. We did not find any significant differences when comparing outcome measures between the eyes that had received ranibizumab or aflibercept immediately prior to the switch. However, this may not be the true representation of the potential level of benefit from the switching from either anti-VEGF agent due to the small sample size. Larger studies will be required to confirm these findings.

In conclusion, an early experience with faricimab in a clinical setting of switched patients is showing stability in the VA and meaningful improvements in the anatomical parameters in a notable proportion. In addition to overall improvement in CST and MV, we observed a significant reduction in CT and serous PED. Although significant improvements were observed in the typical nAMD subgroup, more data are needed particularly to understand the effect on PCV eyes.

## Data availability statement

The raw data supporting the conclusions of this article will be made available by the authors, without undue reservation.

## Ethics statement

This study conformed to the tenets of the Declaration of Helsinki and was approved by the international review board and by the ethics committee. Written informed consent was provided by all participants before participation in the study for the Singapore National Eye Centre (R1921/49/2022). The studies were conducted in accordance with the local legislation and institutional requirements. The participants provided their written informed consent to participate in this study. Written informed consent was obtained from the individual(s) for the publication of any potentially identifiable images or data included in this article.

## Author contributions

FM: Writing – original draft, Writing – review & editing, Conceptualization, Data curation, Formal Analysis, Investigation, Methodology. KT: Conceptualization, Data curation, Formal Analysis, Investigation, Methodology, Writing – original draft, Writing – review & editing. T-ET: Data curation, Writing – review & editing. HC: Data curation, Writing – review & editing. PC: Data curation, Writing – review & editing. S-YL: Data curation, Writing – review & editing. AT: Data curation, Writing – review & editing. RM: Data curation, Writing – review & editing. CMC: Data curation, Writing – review & editing. SS: Data curation, Writing – review & editing. GT: Data curation, Writing – review & editing. IY: Data curation, Writing – review & editing. CMGC: Conceptualization, Data curation, Formal Analysis, Methodology, Supervision, Validation, Writing – original draft, Writing – review & editing.
